# Effect of Persistent Salt Stress on the Physiology and Anatomy of Hybrid Walnut (*Juglans major* × *Juglans regia*) Seedlings

**DOI:** 10.3390/plants13131840

**Published:** 2024-07-04

**Authors:** Jiali Tang, Xinying Ji, Ao Li, Xu Zheng, Yutong Zhang, Junpei Zhang

**Affiliations:** 1 State Key Laboratory of Tree Genetics and Breeding, Research Institute of Forestry, Chinese Academy of Forestry, Beijing 100091, China; tangjl202205@163.com (J.T.); jixinying111@163.com (X.J.); a17637359516@163.com (A.L.); woshizhengxu2022@163.com (X.Z.); 2 College of Resources and Environmental Sciences, China Agricultural University, Beijing 100091, China; yt_vvalker@163.com

**Keywords:** *Juglans*, salt stress, leaf and root anatomy, physiological characteristics, seedling growth

## Abstract

Soil salinization has become one of the major problems that threaten the ecological environment. The aim of this study is to explore the mechanism of salt tolerance of hybrid walnuts (*Juglans major* × *Juglans regia*) under long-term salt stress through the dynamic changes of growth, physiological and biochemical characteristics, and anatomical structure. Our findings indicate that (1) salt stress inhibited seedling height and ground diameter increase, and (2) with increasing salt concentration, relative water content (RWC) decreased, and proline (Pro) and soluble sugar (SS) content increased. The Pro content reached a maximum of 549.64 μg/g on the 42nd day. The increase in superoxide dismutase (SOD) activity (46.80–117.16%), ascorbate peroxidase (APX) activity, total flavonoid content (TFC), and total phenol content (TPC) under salt stress reduced the accumulation of malondialdehyde (MDA). (3) Increasing salt concentration led to increases and subsequent decreases in the thickness of palisade tissues, spongy tissues, leaves, and leaf vascular bundle diameter. Upper and lower skin thickness, root periderm thickness, root diameter, root cortex thickness, and root vascular bundle diameter showed different patterns of change at varying stress concentrations and durations. Overall, the study concluded that salt stress enhanced the antireactive oxygen system, increased levels of osmotic regulators, and low salt concentrations promoted leaf and root anatomy, but that under long-term exposure to high salt levels, leaf anatomy was severely damaged. For the first time, this study combined the anatomical structure of the vegetative organ of hybrid walnut with physiology and biochemistry, which is of great significance for addressing the challenge of walnut salt stress and expanding the planting area.

## 1. Introduction

The total saline–alkali land area is approximately 954 million hectares [1]. A study found that the global saline–alkali area is increasing [2]. By 2050, salinization could lead to a 50% loss of cultivable lands [3]. In China, salt-affected soils constitute 25% of agricultural land, largely distributed in arid and semi-arid zones [4].

Substantial agriculture losses due to salinity, a key abiotic stressor, are evident [5,6]. Current methods, such as developing salt-tolerant varieties or expanding planting areas, can mitigate crop yield impacts [7]. Notably, plant hybrids exhibit enhanced abiotic stress resistance compared to their parents [8], and they are instrumental in China’s economic forest industry [9]. Studies on walnut hybridization have focused on hybrid productivity and disease resistance [10,11,12]. Paradox ‘Zhong Ningyi’, which was developed through artificial hybridization between *Juglans major* and *Juglans regia*, exhibits characteristics such as high affinity with walnut, vigorous growth, growing more than the parents, and resistance to root rot. Although the excellent rootstock variety paradox ‘Zhong Ningyi’ has been widely adopted and cultivated in China, little has been studied regarding its salt stress tolerance.

Soil salinity primarily impairs plant root water uptake, reducing turgor pressure, inducing osmotic stress, and triggering reactive oxygen species (ROS) production, all of which significantly affect plant growth and development [13,14]. Plants counteract salt stress by accumulating osmolytes and enhancing antioxidant capacity, forming physiological and biochemical defense mechanisms [15].

Plants respond to salinity stress with specific anatomical modifications [16]. Leaf structure, reflecting plant adaptation to saline–alkali environments, influences carbon assimilation, water loss, and biomass accumulation [17,18]. Root growth adjustment is crucial for plant survival under salt stress [19]. Salt-stressed plants utilize their root system as a primary stress sensor, triggering structural changes to mitigate stress effects [20]. 

However, plant salt stress sensitivity varies significantly, depending on salt concentration, exposure duration, and growth stage [21]. A study observed an increase in H_2_O_2_ concentration in walnut leaves after salt treatment, resulting in phenolic substance and anthocyanin accumulation, and elevated APX activity [22]. Ji et al. exposed walnuts with four rootstock types to salt stress and found that the MDA content, Pro content, and SS content of each genotype increased by different degrees, and SOD, POD, and CAT enzyme activities showed different patterns of change [23]. Most walnut non-biological stress research has focused on cold and drought resistance, while salt stress research primarily examines physiological and biochemical aspects, with limited reports on salt stress-induced anatomy changes. 

The quality and yield of walnuts are negatively impacted by excessive soil salinity. To expand walnut cultivation and optimize economic returns, we chose to identify low saline–alkali lands for walnut plantations. Thus, our focus was on exploring the responses of growth, physiological and biochemical changes, as well as tissue structure modifications in hybrid walnut (*Juglans major* × *Juglans regia*) seedlings exposed to varying salt concentrations and stress periods. The results provide valuable insights into the mechanisms underlying salt tolerance in hybrid walnut, thus guiding its cultivation in saline soils. 

## 2. Results

### 2.1. Effect of Salt Stress on Seedling Morphological Indicators

**Figure 1:**
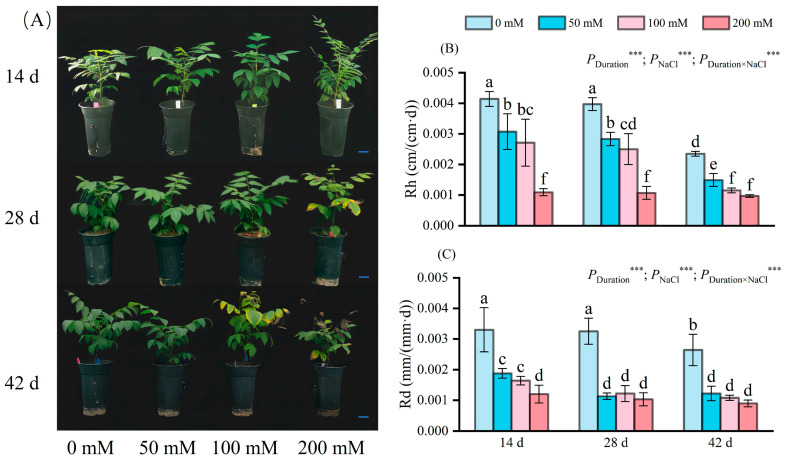
External morphological changes (**A**), the change in Rh (**B**) and Rd (**C**) of seedlings at different NaCl concentrations (0 mM, 50 mM, 100 mM, and 200 mM), on days 14, 28, and 42 of stress are depicted. Scale bar: 5 cm; d indicates the number of days; Rh indicates the average relative growth rates of plant height; Rd indicates the average relative growth rates of stem diameter. The values marked with different letters show significant differences (*p* < 0.05) (Duncan’s test). The error lines indicate the standard deviation of the three replicates; *** means *p* < 0.001, which is the result of a two-way ANOVA.

Symptoms of damage escalated with augmented salt concentrations in the seedlings (Figure 1A). By day 14, no signs of stress were identifiable. However, by day 28, 200 mM NaCl-treated plants showed minor defects, including a handful of shriveled and curled apex leaves. Day 42 witnessed yellowing, shriveling, and curled apices on the upper leaves of the 100 mM treatment group, with leaf drop evident in the 200 mM treatment group.

### 2.2. Effect of Salt Stress on Seedling Growth

#### 2.2.1. Growth Rate of Plant Height (Rh)

With increasing salt concentration, plant height growth exhibited drastic reductions compared to the control levels (Figure 1B). Salt treatment lasting 14 days led to significant decreases in plant height by 25.84%, 34.54%, and 73.67%, respectively, for 50 mM, 100 mM, and 200 mM NaCl groups. At 28 days of stress, the reduction in Rh mirrored the pattern observed at 14 days, albeit with a slightly amplified decline. Following 42 days of stress, the Rh of the plants treated with 50 mM, 100 mM, and 200 mM NaCl solutions continued to decrease, reaching a minimum value of about 0.001 cm/(cm·d). Stress duration, NaCl concentrations, and their interactions have a significant impact on Rh. By day 42, the effects persisted with Rh reaching a minimal level of approximately 0.001 cm/(cm·d). The duration, concentration, and interaction of salt stress significantly influenced Rh.

#### 2.2.2. Growth Rate of Stem Diameter (Rd)

Stem diameter increment (Rd) decelerated with increasing salt level (Figure 1C). After 14 days of salt stress, the Rd of the plants treated with 50 mM, 100 mM, and 200 mM NaCl solution was significantly different from that of the plants in the control group, with reductions of 43.03%, 50.30%, and 63.64%, respectively. Notably, the Rd for 50 mM and 100 mM NaCl plants exceeded that of 200 mM NaCl. By 28 and 42 days, Rd across all treatments was notably diminished compared to the control; the 200 mM NaCl group exhibited the lowest value at approximately 0.001 mm/(mm·d). Stress duration, NaCl concentration, and their interplay significantly influenced Rd. 

### 2.3. Effect of Salt Stress on Seedling Physiological Indicators

#### 2.3.1. Relative Water Content (RWC)

**Figure 2:**
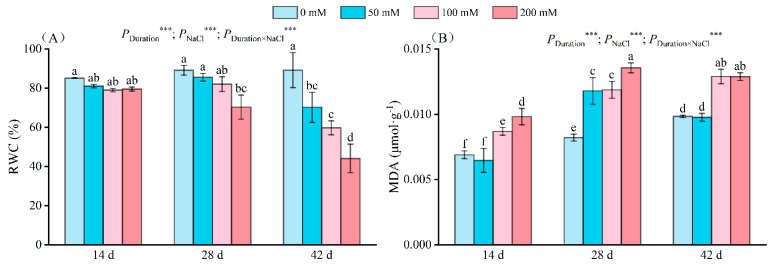
Change in the RWC (**A**) and MDA (**B**) of seedlings at different NaCl concentrations (0 mM, 50 mM, 100 mM, and 200 mM) after 14, 28, and 42 days of stress. RWC indicates the relative water content of leaves, MDA indicates malondialdehyde, and the values marked with different letters show significant differences (*p* < 0.05) (Duncan’s test). The error lines indicate the standard deviation of the three replicates; *** means *p* < 0.001, which is the result of a two-way ANOVA.

As the salt concentration rose, the RWC of the seedlings declined (Figure 2A). After 14 days of salt stress, no significant change in RWC was observed in the salt-treated group. However, after 28 days, the RWC of the seedlings dropped significantly to 70.34% under 200 mM NaCl treatment. After 42 days of salt stress, the RWC of the seedlings treated with 50 mM, 100 mM, and 200 mM salt solutions decreased by 21.27%, 33.02%, and 50.54%, respectively, compared to that recorded for the control group. Stress duration, NaCl concentrations, and their interaction exerted significant influence on RWC.

#### 2.3.2. Malondialdehyde (MDA)

Increased salt levels in seedlings led to enhanced MDA content (Figure 2B). After 14 days of salt stress, the seedlings exposed to 100 mM and 200 mM salt exhibited elevated MDA content by 25.94% and 42.32%, respectively, compared to the controls. By day 28, the MDA content of the 50 mM and 100 mM treatment groups had significantly diverged from the control. Notably, the 200 mM treatment group displayed a distinct MDA level, reaching 0.014 µmol/g; on days 28 and 42, the maximum MDA content under 200 mM salt was 0.013 µmol/g and 0.011 µmol/g, respectively. The duration, NaCl concentration, and their interplay significantly influenced MDA.

#### 2.3.3. Osmotic Adjustment Substances

**Figure 3:**
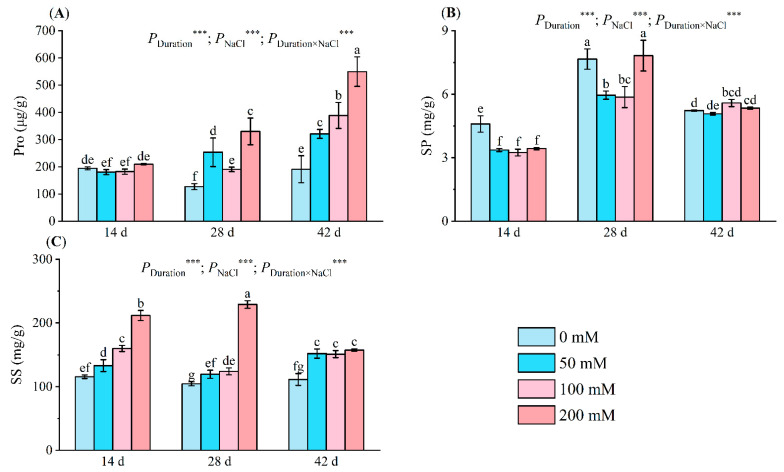
Alterations in Pro (**A**), SP (**B**), and SS (**C**) levels of seedlings at various NaCl concentrations (0 mM, 50 mM, 100 mM, and 200 mM) after 14, 28, and 42 days of stress. Pro indicates proline; SP indicates soluble protein; SS indicates soluble sugar. Distinct letters in the figure represent significant differences (*p* < 0.05), determined by Duncan’s test. Error lines depict the standard deviation of the three replicates, *** means *p* < 0.001, which is the result of a two-way ANOVA.

After 14 days of salt exposure, the proline (Pro) concentration in salt-stressed seedlings remained similar to that in the controls. On days 28 and 42, the seedlings treated with 200 mM NaCl displayed elevated Pro levels—330.17 µg/g and 549.64 µg/g, respectively. Salt treatments of 50 mM and 100 mM on day 28 also differed significantly from the controls. By day 42, the Pro content in these groups had increased by 103.51% and 187.73%, respectively, exhibiting marked differences (Figure 3A).

Following 14 days of salt stress, seedling soluble protein (SP) content noticeably declined under salt treatment, reaching a minimum of 3.24 mg/g at 100 mM, a 29.42% reduction compared to the control. After 28 days, SP content decreased by 22.27% and 23.42% at 50 mM and 100 mM, respectively, but rebounded to the control level (7.82 mg/g) following exposure to a 200 mM salt solution. After 42 days of stress, SP content reached 5.58 mg/g at 100 mM, a 6.88% increase over the control (Figure 3B).

The soluble sugar (SS) content increased significantly under salt stress. Compared with the control, the SS content under 50 mM treatment increased by 14.47–36.42%, while under 100 mM treatment, it increased by 18.85–38.20%. By the 28th day of salt stress, the SS content under 200 mM treatment reached a maximum value of 229.00 mg/g, an increase of 119.26% compared with the control (Figure 3C). The duration of stress, NaCl concentrations, and their interplay greatly affected Pro, SP, and SS.

#### 2.3.4. Antioxidants Substances

**Figure 4:**
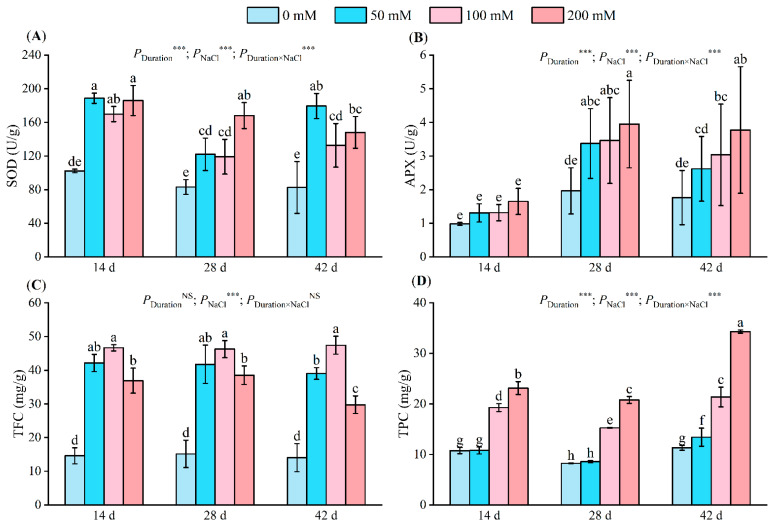
Changes in leaf SOD activity (**A**), APX activity (**B**), TFC (**C**), and TPC (**D**) at different NaCl concentrations (0 mM, 50 mM, 100 mM, and 200 mM) after 14, 28, and 42 days. SOD indicates superoxide dismutase activity; APX indicates ascorbate peroxidase activity; TFC indicates total flavone content; TPC: total phenolic content. The values associated with different letters are significantly different (*p* < 0.05) (Duncan’s test). Error lines show the standard deviation of the three replicates. NS means *p* > 0.05 and *** means *p* < 0.001, which were the results of a two-way ANOVA.

Following a 14-day salt stress period, superoxide dismutase (SOD) activity significantly diverged in the salt treatment groups compared to the control, reaching values of 188.69 U/g, 169.77 U/g, and 185.88 U/g, representing an increment of 84.47%, 65.98%, and 81.17%, respectively. After 28 days, SOD activity escalated with increasing salt concentration, peaking at 168.00 U/g at 200 mM, indicating a rise of 102.04%. After 42 days, the SOD activity in the 50 mM treatment group reached 179.40 U/g, signifying an increase of 117.16%. The SOD activity in the 100 mM and 200 mM treatment groups decreased marginally compared to the 50 mM group (Figure 4A).

Ascorbate peroxidase (APX) activity increased with an increase in the salt concentration. After 14 days of salt stress, there was no significant difference in APX activity under salt treatment compared with the control group. After 28 days of stress, the APX activity was significantly different between the salt treatments and the control. In the 200 mM treatment, the APX activity reached 3.94 U/g and 3.77 U/g on days 28 and 42 (Figure 4B).

Total flavone content (TFC) first increased and then decreased with an increase in salt concentration (Figure 4C). A significant difference occurred between each salt treatment and the control; all treatments mentioned below reached their respective highest values at 100 mM, with values of 46.64 mg/g, 46.25 mg/g, and 47.39 mg/g being 3.19 times, 3.05 times, and 3.38 times higher than those recorded for the control, respectively. After 42 days of stress, the TFC of the 50 mM treatment group reached 39.04 mg/g, which was significantly lower than that of the 100 mM treatment group but higher than that recorded for the 200 mM treatment group (by 1.31 times).

**Figure 5:**
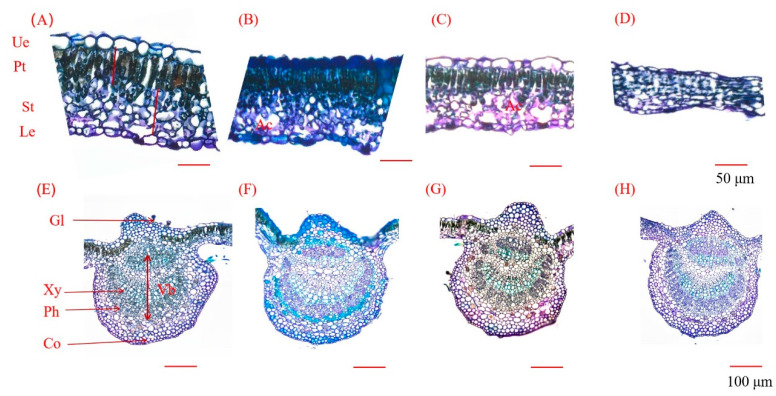
Leaf anatomical structure after 42 days of salt stress. Vb: vascular bundle; Gl: glandular trichome; Xy: xylem; Ph: phloem; Co: collenchyma; Ue: upper epidermis; Le: lower epidermis; Pt: palisade tissue; St: spongy tissue; Ac: aerenchyma. (**A**,**E**): 0 mM salt treatment; (**B**,**F**): 50 mM salt; (**C**,**G**): 100 mM salt treatment; (**D**,**H**): 200 mM salt treatment. (**A**–**D**): Leaf blade tissue structure; scale bar: 50 µm. (**E**–**H**): Cross-sectional tissue structure of leaf veins; scale bar: 100 µm.

Total phenolic content (TPC) increased with an increase in salt concentration (Figure 5D). All treatments reached their respective highest values at 200 mM, with 23.15 mg/g, 20.79 mg/g, and 34.29 mg/g representing 2.15 times, 2.52 times, and 3.03 times the control’s TPC, respectively. Within the same stress period, no significant difference existed between the control and the 50 mM treatment groups. However, significant TPC disparities were noted between the control and other treatment groups. Stress duration, NaCl concentrations, and their interactions significantly influenced SOD, APX, TFC, and TPC. 

### 2.4. Effect of Salt Stress on the Seedling Leaf Micro-Morphoanatomical Characteristics

#### 2.4.1. Palisade Tissue

**Figure 6:**
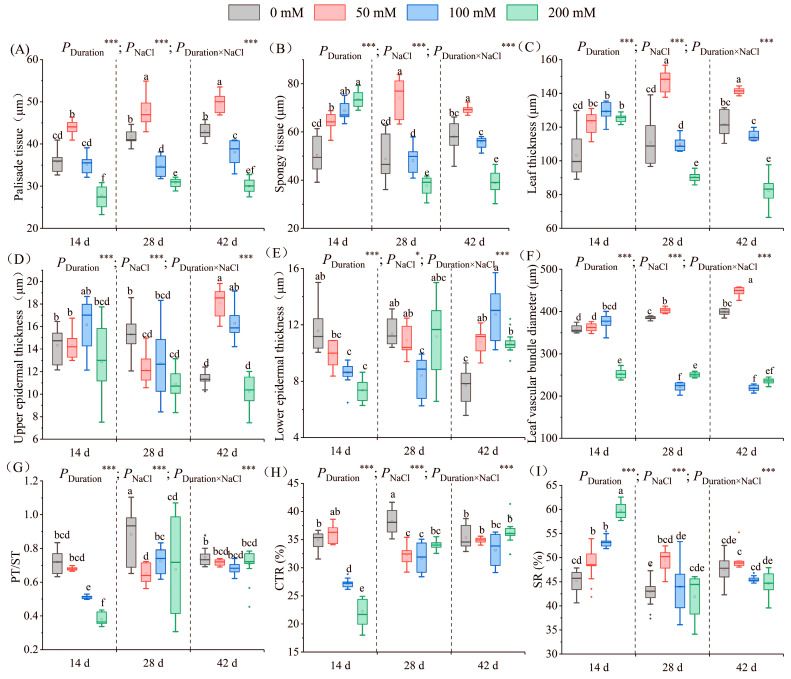
Leaf parameters under salt treatment. The changes in the palisade tissue thickness (**A**), spongy tissue thickness (**B**), leaf thickness (**C**), upper epidermis thickness (**D**), lower epidermis thickness (**E**), vascular bundle diameter (**F**), PT/ST (**G**), CTR (**H**), and SR (**I**) of seedlings at different NaCl concentrations (0 mM, 50 mM, 100 mM, and 200 mM) are illustrated after stress for 14, 28, and 42 days. PT/ST indicates the ratio of palisade tissue thickness to spongy tissue thickness, CTR indicates cell tightness ratio, and SR indicates leaf structure looseness. Different letters within the same stress period indicate significant differences (*p* < 0.05) (Duncan’s test). The error lines represent the standard deviation from the three replicates. Significance levels *, *** represent *p* < 0.05 and *p* < 0.001, representing the outcomes of a two-way ANOVA.

As the salt concentration escalated, palisade tissue thickness initially increased and subsequently diminished (Figure 5A–D). Over the three designated stress periods, the maximum palisade tissue thickness post-treatment with 50 mM salt solution was 43.84 µm, 47.99 µm, and 49.72 µm, representing a 22.49%, 14.94%, and 15.68% increase on days 14, 28, and 42, respectively. The 200 mM treatment group exhibited the thinnest thickness of 27.74 µm, 30.95 µm, and 30.01 µm, representing a 22.49%, 25.87%, and 30.18% decrease on days 14, 28, and 42, respectively. After 14 days of salt stress, the thickness post-treatment with 100 mM salt solution equaled that in the control, but after 28 and 42 days of stress, the palisade tissue thickness post-treatment with 100 mM salt solution diverged significantly from that in the control, attaining 34.69 µm and 38.08 µm, respectively (Figure 6A).

#### 2.4.2. Spongy Tissue

After 14 days of stress, the spongy tissue thickness increased with the escalating salt concentration. The thickness differed considerably between each salt concentration treatment and the control. The peak thickness (73.46 µm) was noted following treatment with 200 mM salt solution, indicating a marked 45.95% increase versus the control. After 28 and 42 days of salt stress, the change in spongy tissue thickness initially augmented and subsequently diminished. The thickness after treatment with 50 mM and 200 mM salt solution was significantly different from that recorded in the control. After 28 days of stress, the highest thickness observed was 74.02 µm in the 50 mM treatment group, signifying a 51.68% increase versus the control. After 42 days of stress, the thickness post-treatment with 200 mM salt solution exhibited the most substantial decrease; it was merely 38.92 µm, representing a 32.92% decrease versus the control (Figure 6B).

#### 2.4.3. Leaf Thickness

As the salt concentration increased, the leaf thickness first increased and then decreased. After 14 days, the leaf thickness in the salt groups significantly outpaced the control values; the measurements were 122.47 µm, 129.39 µm, and 125.09 µm post 50, 100, and 200 mM NaCl treatments, respectively. After 28 days, the 50 mM treatment exhibited a 33.24% increase compared to the control. By day 42, the 50 mM treatment group had the highest thickness, at 141.48 µm, while the 200 mM treatment group had the lowest, at 82.53 µm (Figure 6C).

#### 2.4.4. Upper Epidermal Thickness

After 14 days of salinity stress, the upper epidermal thickness initially augmented and subsequently subsided with escalating salt concentration. The thickness was 16.14 µm after treatment with 100 mM NaCl, signifying an increase of 12.53% compared to the control group. Subsequently, the thickness decreased to 12.87 µm post 200 mM salt treatment, indicating a decrease of 10.25%. After 28 days of stress, the upper epidermal thickness was notably lower than in the control group, recording values of 12.20 µm, 12.75 µm, and 10.90 µm for the respective treatment groups. After 42 days of stress, the upper epidermal thickness fluctuated again with salt concentration (Figure 5A–D). The highest thickness of 18.07 µm was observed in the 50 mM treatment group, representing a 1.59-fold increase over the control group. The lowest thickness of 10.25 µm was recorded in the 200 mM treatment group (Figure 6D).

#### 2.4.5. Lower Epidermal Thickness

Following 14 days of salinity exposure, the lower epidermal thickness decreased progressively with increasing salt concentration. The lowest value of 7.35 µm was recorded following the 200 mM treatment, representing a 32.31% decrease from the control group. After 28 days, the lower epidermal thickness initially declined and subsequently increased with salt concentration. The 100 mM treatment group exhibited a significant difference, recording the lowest value of 8.38 µm, a 21.18% reduction from the control group. After 42 days, the lower epidermal thickness initially rose and subsequently declined with salt concentration (Figure 5A–D). All salt treatment groups displayed a significant increase in thickness compared to the control group. The 100 mM treatment group exhibited the highest value of 12.73 µm, 1.65 times greater than the control group (Figure 6E).

#### 2.4.6. Leaf Vascular Bundle Diameter

The vascular bundle diameter first increased and then decreased with an increase in salt concentration. After 14 days of salt stress, the diameter in the 200 mM treatment group significantly diverged from the others, reaching a minimum of 256.45 µm, representing a 28.53% reduction compared to the control group. After 28 days of salt stress, the diameter in the 100 mM treatment group showed the lowest value of 221.97 µm, which was 0.78 times lower than that recorded in the control group. After 42 days of salt stress, the diameter of the vascular bundles differed significantly between each treatment group and the control group (Figure 5E–H). The diameter reached the highest value of 447.80 µm in the 50 mM treatment group, and it reached the lowest value of 218.91 µm in the 100 mM treatment group (Figure 6F).

#### 2.4.7. Ratio of Palisade Tissue Thickness to Spongy Tissue Thickness (PT/ST)

After 14 days of salt stress, PT/ST decreased with an increase in salt concentration. The ratio in the 200 mM treatment group showed the lowest value of 0.38, which was 47.31% lower than that in the control group. After 28 and 42 days of stress, PT/ST first decreased and then increased with an increase in salt concentration. A significant difference in PT/ST was recorded between each salt treatment group and the control group on day 28, indicating a decrease of 26.03%, 17.62%, and 23.38%, respectively. After 42 days of stress, the ratio in the 100 mM treatment group had the lowest value of 0.68 (Figure 6G).

#### 2.4.8. Leaf Cell Tightness Ratio (CTR)

Following 14 days of stress, CTR initially elevated but subsequently declined with increasing salt concentration. Noticeable CTR variations were recorded among the 100 mM and 200 mM treatment groups and the control group, declining by 21.97% and 36.22%, respectively. After 28 days of stress, significant CTR differences existed among each salt treatment group and the control group, decreasing by 12.98%, 15.87%, and 12.38% for the 50 mM, 100 mM, and 200 mM salt treatments, respectively. After 42 days of stress, CTR initially fell but subsequently rose with increasing salt concentration. The CTR in the 100 mM treatment group exhibited the lowest value (Figure 6H).

#### 2.4.9. Leaf Structure Looseness (SR) 

After 14 days of salt stress, SR escalated with salt concentration. A significant difference in SR was recorded between each salt treatment group and the control group. The 200 mM treatment exhibited the highest value of 58.70%, 1.2 times greater than the control. After 28 and 42 days, SR initially rose and subsequently declined with salt concentration. The peak SR was observed at 50 mM (Figure 6I). Stress duration, NaCl concentrations, and their interplay significantly influenced leaf parameters.

### 2.5. Effect of Salt Stress on Seedling Root Micro-Morphoanatomical Characteristics

#### 2.5.1. Root Diameter

**Figure 7:**
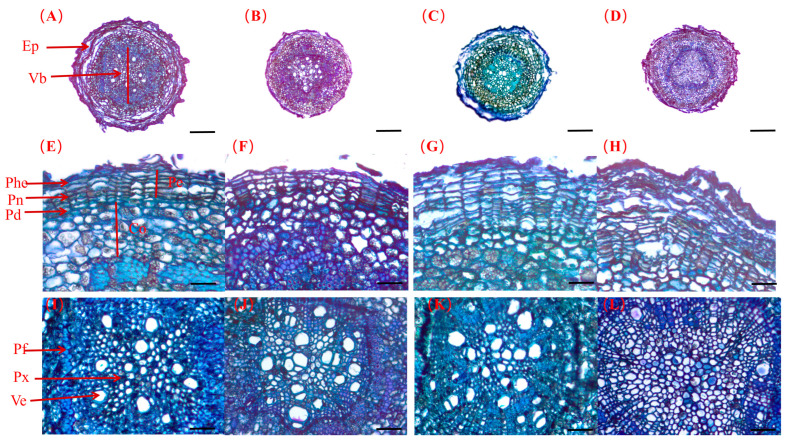
The microscopic anatomy of primary lateral roots across varying salt concentrations (0 mM, 50 mM, 100 mM, and 200 mM) up to a stress duration of 42 days (**A**–**L**). (**A**,**E**,**I**) represent 0 mM salt treatment; (**B**,**F**,**J**) represent 50 mM salt treatment; (**C**,**G**,**K**) represent 100 mM salt treatment; (**D**,**H**,**L**) represent 200 mM salt treatment. Ep: epidermis; Vb: vascular bundle; Pe: pericycle; Co: cortex; Phe: phellem; Pn: phellogen; Pd: phelloderm; Pf: phloem fibers; Px: primary xylem; Ve: vessel element. (**A**–**D**): The cross-section tissue structure of roots; scale bundle: 100 µm. (**E**–**H**): The epidermal tissue microstructure; scale bundle: 25 µm. (**I**–**L**): Vascular bundle microstructure; scale bundle: 25 µm.

**Figure 8:**
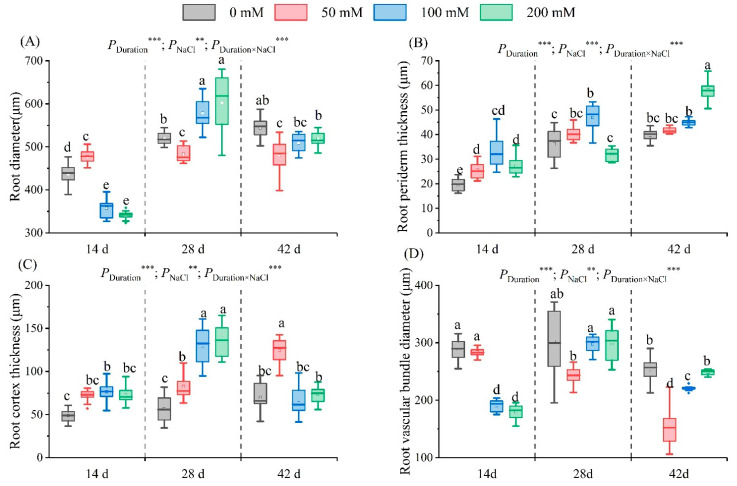
Root parameters under salt treatments. The change in the root diameter (**A**), root periderm (**B**), root cortex (**C**), and root vascular bundle diameter (**D**) of seedlings at different NaCl concentrations (0 mM, 50 mM, 100 mM, and 200 mM) is illustrated after 14, 28, and 42 days of stress. The values marked with different letters in the figure indicate significant differences (*p* < 0.05) (Duncan’s test). The error lines indicate the standard deviation of the three replicates. Significance levels ** and *** represent *p* < 0.01 and *p* < 0.001, representing the outcomes of a two-way ANOVA.

After 14 days of salt stress, the root diameter first increased and then decreased with an increase in salt. A maximum diameter of 470.90 μm was observed in the 50 mM treatment regime. Conversely, a minimum of 341.16 µm was detected in the 200 mM treatment group, a 21.78% reduction relative to the control’s value. After 28 and 42 days of stress, the root diameter first decreased and then increased with an increase in the salt concentration (Figure 7A–D). The lowest diameters were recorded in the 50 mM treatment group (482.69 μm and 479.36 µm for 28 and 42 days, respectively), representing a decrease of 6.80% and 11.89% compared to the values recorded in the control group. After 28 days, the diameter increased by 11.68% and 15.99%, respectively, in the 100 mM and 200 mM treatment regimes. However, following 42 days of stress, the diameter declined (Figure 8A).

#### 2.5.2. Root Periderm Thickness

Subsequent to 14 and 28 days of salt stress, the root periderm thickness first increased and then decreased as the salt concentration augmented. The maximum thickness was recorded in the 100 mM treatment, amounting to a 68.16% and 72.00% increase over the control. The thickness in the 50 mM and 200 mM treatment groups rose by 31.49% and 40.80%, respectively, compared to the control. After 42 days of stress, the thickness increased with salt concentration (Figure 7E–H). Notably, in the 200 mM treatment group, a substantial increase in thickness (46.49%) was observed compared to the control group (Figure 8B).

#### 2.5.3. Root Cortex Thickness

Following 14 days of salt stress, root cortex thickness initially increased and then decreased with increasing salt concentration. The peak value was observed at 100 mM, exceeding the control by 56.53%. On day 28, increases in thickness were observed as the salt concentration rose, showing significant differences from the control group. The greatest thickness (134.52 μm) occurred at 200 mM, which was 2.36 times that recorded in the control group. By day 42, cortex thickness again exhibited an initial increase and then decrease with increasing salt concentration (Figure 7E–H). A peak thickness of 124.35 μm was observed in the 50 mM treatment group, and it was 1.77 times higher than the thickness recorded in the control group; it was also significantly higher than that recorded in the other treatment groups (Figure 8C).

#### 2.5.4. Root Vascular Bundle Diameter

After 14 days of salt stress, the vascular bundle diameter declined with increased salt concentration. The diameter of the 200 mM treatment had the lowest value of 179.27 μm, a 37.90% reduction from the that of the control. After 28 and 42 days of stress, the diameter initially reduced, but subsequently rose (Figure 7I–L). The lowest values of 242.86 μm and 152.91 µm, respectively, were observed in the 50 mM treatment group, representing a 19.48% and 39.56% reduction from the control (Figure 8D). Stress duration, NaCl concentration, and their interactions significantly influenced root parameters.

### 2.6. Comprehensive Analysis

#### 2.6.1. Correlation Analysis

**Figure 9:**
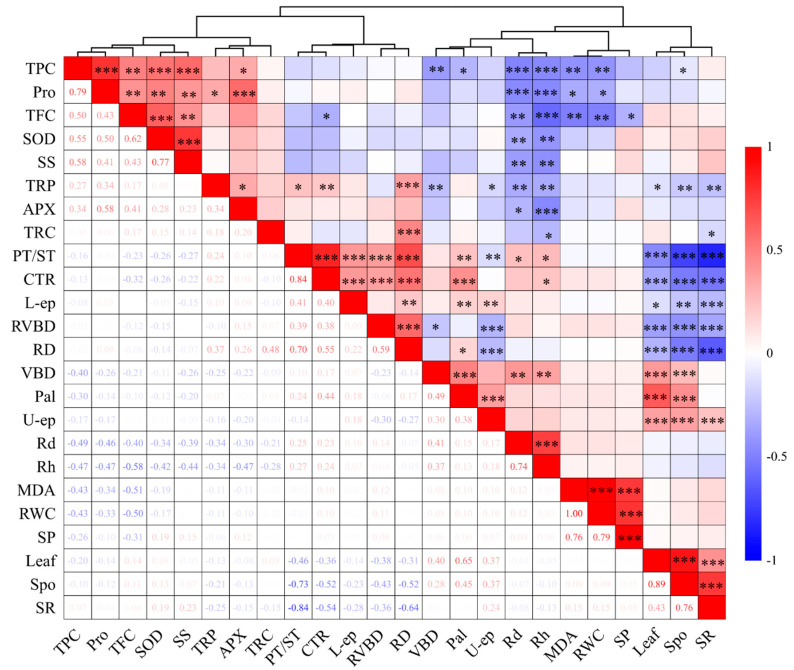
The correlation coefficients and hierarchical cluster analysis for salt stress parameters. Red represents positive correlation, and blue represents negative correlation. The darker the color, the more significant the correlation; * *p* < 0.05, ** *p* < 0.01, *** *p* < 0.001. Rh: mean relative growth rate of seedling height; Rd: mean relative growth rate of ground diameter; RWC: relative water content; MDA: malondialdehyde; Pro: proline; SP: soluble protein; SS: soluble sugar; SOD: superoxide dismutase; APX: ascorbate peroxidase; TPC: total phenolic content; TFC: total flavonoid content; Spo: spongy tissue thickness; Pal: palisade tissue thickness; U-ep: upper epidermis thickness; L-ep: lower epidermis thickness; Leaf: leaf thickness; VBD: vein bundle diameter; CTR: cell tightness ratio; SR: tissue looseness; PT/ST: fence–sea ratio; RD: root diameter; TRC: root cortex thickness; RVBD: root vascular bundle diameter; TRP: root periderm thickness.

To ascertain the correlation among the indicators, Pearson’s correlation analysis and cluster analysis were performed (Figure 9). Rd and Rh exhibited notable negative correlations with TPC, Pro, TFC, SOD, SS, TRP, and APX, and significant positive correlations with leaf vascular bundle diameter and PT/ST. TPC and TFC demonstrated significant negative correlations with MDA and RWC, as well as significant positive correlations with SOD and SS. Leaf spongy tissue thickness, leaf thickness, and leaf SR showed significant negative correlations with root diameter, root vascular bundle diameter, PT/ST, leaf CTR, and lower epidermis thickness, and leaf spongy tissue thickness and leaf thickness showed significant positive correlations with leaf SR, leaf vascular bundle diameter, leaf palisade tissue thickness, and upper epidermis thickness. Root diameter and root vascular bundle diameter showed significant positive correlations with leaf CTR and PT/ST. The indicators were grouped by the hierarchical clustering method, resulting in three main clusters: the first cluster included leaf osmotic regulatory substances and anti-reactive oxygen species; the second cluster consisted of the anatomical structure of leaves and roots; the third cluster included leaf physiology, anatomical indicators, and growth indicators.

#### 2.6.2. Principal Component Analysis

**Figure 10:**
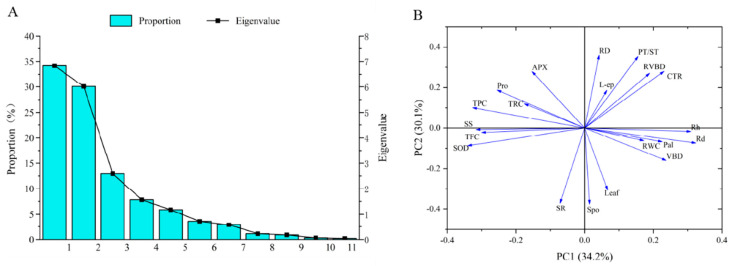
Results of principal components analysis (PCA). (**A**) Variance explained and eigenvalues for each principal component. (**B**) Parameter distribution from PCA as well as their relative impact on PC1 and PC2.

Several plant indicators exhibit distinct mechanisms for salt tolerance, potentially biasing evaluations using a singular indicator. This study employed principal component analysis of 20 indicators from salt-stressed seedlings across three treatment periods. The results indicated that the cumulative contribution rate of the first five principal components reached 90.92%, encapsulating substantial indicator parameter information (Figure 10). The foremost component contributed 34.18%. SOD, TPC, Rd, SS, and Rh, with the highest feature values, significantly affected the first principal component. The second principal component, accounting for 30.10% of the contribution, was significantly impacted by Spo, SR, RD, and PT/ST indicators. 

## 3. Discussion

### 3.1. Effect of Salt Stress on Seedling Indicators

Exterior morphology and growth status are key indicators of plant salt damage severity [24]. Leaf-tip necrosis is the initial indication of NaCl stress-induced injury [25]. Salt stress manifests in leaf symptoms such as yellowing, scorching, and shedding (Figure 1), similar to symptoms in groundcherry [26] and mango [27] under NaCl stress. The seedling height, ground diameter, and biomass of plants, such as pistachio [28], tea [29], Makino [30,31], citrus [32], and red amaranth [33] are suppressed. Our study found that salt stress significantly inhibits seedling growth, particularly at high salt levels. These alterations may be linked to increased energy expenditure in the salt environment to maintain ion balance and repair salt stress-induced damage. In this study, seedling growth was negatively correlated with osmoregulatory substances and anti-reactive oxygen species in the leaves (Figure 9).

### 3.2. Effect of Salt Stress on Physiological Indicators of Seedling Leaves

The relative water content accurately reflects the equilibrium between water absorbed by a plant and water lost through transpiration [34]. Decreased plant relative water content under stress results from (i) diminished water accessibility to roots; (ii) constricted root activity and water acquisition; (iii) disruption in membrane stability and augmentation in electrolyte leakage, leading to water loss; and (iv) reduced soil water potential [35]. Our findings indicate a decline in leaf relative water content with escalating salt concentration and stress duration (Figure 2A). These trends are also seen in tomato [36] and black locusts [37].

Malondialdehyde serves as a crucial physiological marker reflecting cellular membrane integrity. Modifications in MDA content reflect the extent of environmental damage to plants and their stress resilience [38,39]. This study revealed that malondialdehyde content did not significantly differ at low salt levels during the initial phase of stress, suggesting minimal membrane damage [40]. However, with escalating salt concentration and stress duration, MDA content escalated significantly, indicating membrane disruption. Notably, at high salt levels, leaf tissue boundaries became indistinct, and the membrane system was compromised (Figure 5D). Similar results were observed for grafted grapevine, mango [27], and Cornus [41]. Zhang et al. discovered that in barbary wolfberry, the variations in CAT, SOD, and POD activities mirrored those of MDA content under salt treatment [42]. 

Osmoprotectants are pivotal for preserving salt tolerance during salinity stress [43]. Accumulation of osmolytes and compatible solutes under salt stress offers a tolerance mechanism by shielding essential macromolecules from oxidative damage [44]. Research indicates that proline accumulation is a prevalent physiological response in numerous plants under abiotic stress, signifying stress resistance [44]. Proline levels escalated with escalating NaCl concentration in chili pepper, kiwifruit [45], and banana [46]. Our findings revealed that proline production at elevated salt concentrations was 2.87 times greater than in the control group (Figure 3A). The salt-induced proline surge may be due to an accelerated protein hydrolysis rate as protein synthesis shifts towards proline accumulation. A decelerated degradation rate may also contribute to the high proline content [47]. Proline fortifies the plasma membrane by augmenting diverse antioxidant systems and mitigating membrane lipid and protein oxidation due to salinity-induced oxidative stress [48]. In our study, proline demonstrated a substantial positive correlation with APX activity and SOD activity (Figure 9). Initially, proline content declined slightly. This could be attributed to the seedlings’ capacity to maintain cell osmotic potential via lower proline content at low NaCl concentrations. Compared to synthetic organics, ions are more efficient for osmotic potential adjustment [15].

Plant protein accumulation under saline conditions may act as nitrogen storage for reuse after stress mitigation. These proteins might also contribute to osmotic regulation [49]. Studies on kenaf [50] and oats [51] have demonstrated an increase in soluble protein concentration in seedlings under salinity stress. However, these findings contradict our study, revealing a substantial decrease in soluble protein content under salt stress (Figure 3B). Baniasadi et al. suggested that salt stress-induced protein reduction may result from enzyme denaturation and reduced amino acid accessibility [52]. Other studies have proposed that under mild NaCl stress, plants mitigate stress damage through protein hydrolysis [53,54]. Concurrently, the stable soluble protein content observed in the latter stages of our study may correlate with the substantial increase in proline, protecting proteins from degradation and denaturation.

Under harsh conditions, soluble sugars can serve as regulators of infiltration, simultaneously providing organic matter for normal plant growth [55]. Evaluation through Figure 3C reveals a rapid rise in soluble sugar concentration. Synergistic interplay between the solutes and proline may stimulate the biosynthesis of stress-alleviating plant metabolites, preserving osmotic balance. This balance maintains cellular integrity, ensuring membrane firmness and protection, thereby preventing oxidative stress and photo-oxidation in stressed plants [56]. In this study, proline and solutes play pivotal roles in osmotic regulation, with soluble proteins primarily hydrolyzed into amino acids to mitigate damage.

Salt stress elicits ROS accumulation, resulting in oxidative stress-induced plant toxicity [57]. Plants mitigate these damages via antioxidant defenses, comprising both enzymatic and non-enzymatic mechanisms for ROS scavenging and detoxification [58]. SOD is the first line of defense against ROS in plants [59,60]. Here, we observed a rise initially, followed by a decrease in SOD activity with increasing salt concentration (Figure 4A). Findings consistent with this pattern have been reported in pistachio [61] and groundcherry. A diminished SOD activity might stem from a reduced SOD activity-associated protein content or structural disruption. However, we also discovered that by day 28 of salt stress, the increase in SOD enzyme activity was lower than that on the 14th day of stress, which was related to the significant increase in APX activity and proline content. APX, a crucial antioxidant enzyme in active oxygen metabolism pathways in plants and animals, aids in H_2_O_2_ removal from chloroplasts and vitamin C metabolism [62,63,64]. In some studies, mangroves [64] and kiwifruit [45] were reported to have higher APX activity in highly saline environments, which matched the results of this study. Phenolic compounds, the primary group of secondary metabolites, aid in free radical scavenging, singlet oxygen quenching, and peroxide decomposition under biotic and abiotic stress conditions [65]. Total flavonoid and polyphenol content exhibited a significant negative correlation with malondialdehyde content (Figure 9). Phenolic compounds exert antioxidant activity by either inactivating lipid free radicals or preventing hydroperoxides from decomposing into free radicals [66]. In this study, the total phenol and flavone content increased with an increase in salt stress (Figure 4C,D), and similar results were recorded in studies on kenaf [50], tomato [36], almond [67], and pea [68].

### 3.3. Effects of Salt Stress on the Microstructure of Seedling Leaves

Palisade tissue thickness in leaves mirrors the effects of environmental factors on plants’ adaptation, indicating an adaptive response to specialized habitats [69]. Research indicates that thicker palisade tissue under salinity stress enhances moisture retention, prevents physiological drought from salt stress, boosts light energy capture by leaves, and promotes organic matter synthesis for plant metabolism. This helps adaptation to salt-stress environments [70]. Our study showed that palisade tissue thickened under low salt concentrations, contributing to normal leaf physiological activity. However, as salt concentration and stress duration escalated, spongy and palisade tissue thickness diminished (Figure 6). This serves as a defensive mechanism against water scarcity under salt stress, reducing mechanical damage and transpiration [71]. Leaf structure tightness reflects spongy tissue development and can induce leaf spongy tissue degradation in plants grown in high-salt soil [72], aligning with our findings. At high salt concentrations, spongy tissue size decreased due to water loss, with some structures appearing disrupted or disintegrated (Figure 5D). The abnormal tissue structure suggests that cell structure changes under high salt concentrations, inhibiting leaf physiological activities.

Similarly, this research detected an increase in leaf thickness and spongy tissue at low salt concentrations accompanied by a decline in the PT/ST ratio (Figure 6), echoing findings from zucchini squash research [73]. Interestingly, the leaf structure constriction initially heightened under low salt stress. This is significant, as greater leaf structure constriction can enhance CO_2_ diffusion in the mesophyll, offsetting salt stress-induced stomatal constraints [74]. These salt-induced morphological modifications facilitate enhanced transport of CO_2_ to the mesophyll as the stress duration extends [75]. During the latter part of this research, leaf tissue porosity and tissue tightness exhibited reduced sensitivity to salt stress, potentially due to the leaf’s robust adaptability and salt tolerance via structural characteristic adjustments.

In our study, we observed an increase in leaf thickness during the pre-stress period, attributable primarily to the amplified thickness of palisade tissue. This finding was similar to those of *Lycium barbarum* L. [76] and kiwifruit [77]. Conversely, the leaf thickness significantly decreased during the latter stages of stress, mirroring findings for *Hibiscus moscheutos* [78], *Passiflora* L. [79], and *Curcuma longa* L. [80] under salt stress. This reduction was primarily due to the diminished thickness of spongy tissue. Some research indicates a correlation between leaves. Thinning of leaves is primarily related to spongy tissue thinning. Research indicates a correlation between leaf thickness and Na^+^ content, a possible manifestation of salt excretion when salinity surpasses the leaf threshold for vacuolar accumulation [81,82]. 

In advanced salt stress stages, lower leaf epidermal thickness reduced (Figure 5 and Figure 6), potentially acting as a salt stress coping mechanism. The growth of fenestrated tissues may be reduced to enhance intercellular spaces and offset transpiration reduction [83], mirroring findings from *Guettarda speciosa* [71]. In this study, leaf epidermal thickness remained consistent or increased under salt treatment. Research indicates that increased epidermal thickness better mitigates leaf surface water loss and enhances water retention efficiency [84,85]. Furthermore, some studies suggest that epidermal thickening facilitates the sequestration of Na^+^ by accumulating high levels of Na^+^ in epidermal cells while maintaining low levels in mesophyll cells [86].

Plant vascular systems are conduits for nutrients, ions, water, and hormones [87]. Xylem transports water and nutrients, while phloem conveys photosynthates and signaling molecules [88]. Leaf vein thickness increased in *Lycium barbarum* L., enhancing water/nutrient absorption and retention and raising relative water content [76]. However, in this study, the diameter of the vascular bundles decreased significantly under high salt concentrations (Figure 6). The reduction in the vascular bundle diameter decreases the efficiency of Na^+^ transport from root to leaf, which in turn decreases the accumulation of Na^+^ in leaf tissue [89]. Furthermore, some studies suggest that epidermal thickening facilitates the sequestration of Na^+^ by accumulating high levels of Na^+^ in epidermal cells while maintaining low levels in mesophyll cells.

### 3.4. Effect of Salt Stress on the Microstructure of Seedling Root Tissues

Some studies have shown that the growth and development of underground parts are promoted in a low-salt environment [19,90,91]. We found similar changes in this study, where low salt concentrations promoted an increase in seedling root diameter. Root development enhances the amount of nutrients available to plants, which promotes an increase in plant mass under low salt stress [92]. However, root diameter decreased during the middle and late stress stages, similar to grapevine studies [31]. The reduction in root cross-sectional diameter helps to maintain turgor pressure more effectively in the absence of water, as the reduction in the size and number of root cells effectively increases water potential [93,94].

Salt stress can modify vascular tissue dimensions to restrict water loss, augment conduction, and sustain transportation for superior conduction of water, essential nutrients, photosynthetic products, and assimilation relocation [87]. This investigation revealed a significant decrease in the vascular bundle diameter during the pre-stress phase, specifically under 50 mM (Figure 7). Diminished vascular bundle diameter aids in limiting Na^+^ transport in the root system. Nevertheless, the reduction in root vascular bundle diameter diminished at 100 and 200 mM (Figure 8). The partial enhancements evident in the root vascular bundle suggest a plant strategy to enhance water use efficiency and Na^+^ exclusion in the shoot via Na^+^ partition assimilation [95,96].

Increased salinity may amplify epidermal thickness and catalyze lysogenic aerenchyma development. These features mitigate Na^+^ influx and protect the plant [95,97,98]. Correspondingly, our findings indicate a significant augmentation in root cortex thickness and an augmentation of the root periderm in the mid- and late stages of stress (Figure 7). Zhang et al. demonstrated that salt ions can be sequestered from the periderm, significantly restricting water transport and lateral diffusion [99]. The same effect was also found in maize, mung beans, and peas [68]. However, at higher stress levels, cortical thickness does not significantly increase under 100 mM and 200 mM salt concentrations (Figure 8). This could be due to nutrient depletion and leaf tissue damage, impeding organic matter production and transportation (Figure 5), thereby hindering root cortex expansion [100]. Moreover, at a 200 mM salt concentration, the root cortex and periderm tissue deform and collapse (Figure 8). Our study revealed that at high salt concentrations, a reduction in vascular bundle diameter can mitigate salt damage by limiting salt ascent, and reducing Na^+^ influx by augmenting cortex thickness.

## 4. Materials and Methods

### 4.1. Plant Materials and Growth Conditions

In October 2021, we collected the seeds of 20-year-old hybrid walnut (*Juglans major* × *Juglans regia*) from the Chinese Academy of Forestry Walnut Germplasm Resource Library located in Luoning County, Henan Province (34°21′ N, 111°28′ E; altitude: 581 m) to conduct this study. The experiment was conducted at the Chinese Academy of Forestry, China’s greenhouse (40° N, 116°140′ E; 61 m altitude), which maintained an average temperature of 24.47 °C. On 1 May 2022, the three-month-stored seeds were sown in plastic pots (18 cm diameter, 25 cm height) with one seed per pot. The substrate was a 1:1 *v*/*v* mix of peat soil and pastoral soil. Seedlings were managed routinely throughout their growth.

### 4.2. Experimental Design

**Figure 11:**
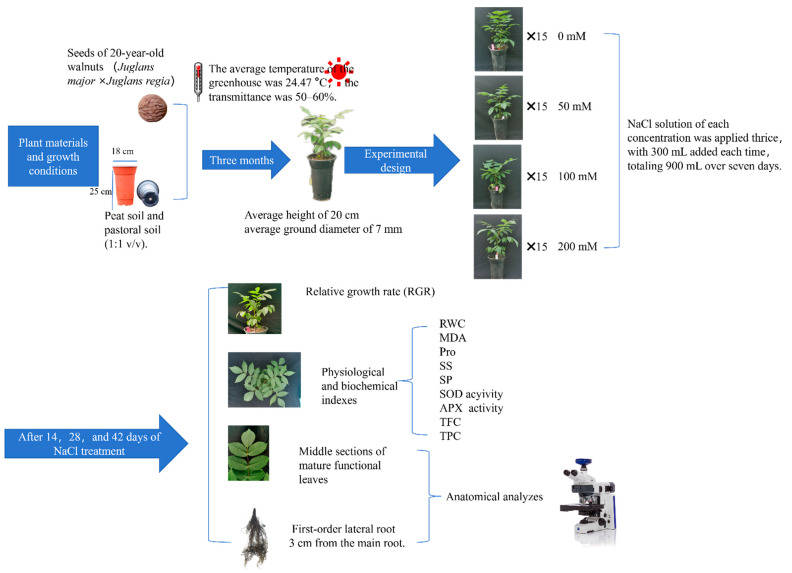
Experimental design and methodology.

By 1 August 2022, each seedling had reached an average height of 20 cm and an average ground diameter of 7 mm. Healthy seedlings displaying consistent growth were subjected to one of four NaCl concentrations (0 mM, 50 mM, 100 mM, and 200 mM) for 42 days. Each concentration had three replicate groups, with five seedlings per replicate. To minimize the impact of the salt solution, NaCl solution of each concentration was applied thrice, with 300 mL added each time, totaling 900 mL over seven days. The NaCl used in the salt solution was dissolved in 1/2 strength Hoagland nutrient solution, proportional to the corresponding weight. A 1/2 strength Hoagland nutrient solution devoid of NaCl was administered every seven days for nutrient supply. To maintain consistent salt levels in each pot, a sufficiently large plastic tray was placed beneath each pot to capture solution runoff from water and nutrient medium replenishment. After 14, 28, and 42 days of NaCl treatment, growth indices were measured, and functional leaves from the east, south, west, and north orientations, as well as the first lateral underground roots, were collected to assess physiological, biochemical, and histological characteristics (Figure 11).

### 4.3. Determination of Indices

#### 4.3.1. Relative Growth Rate (RGR)

Healthy seedlings randomly chosen from each group and numbered were monitored for height (centimeters, determined via measuring tape) and ground diameter (millimeters, identified through a vernier caliper). These measurements were taken on days 0, 14, 28, and 42 after salt stress exposure. The average relative growth rates of plant height (Rh) and stem diameter (Rd) were calculated using the formulae presented below [101].

Rh=(lnQH−lnQH0)/Δt

(QH indicates seedling height, QH0 indicates the seedling height on day 0, and Δt indicates salt stress days)

Rd=(lnQD−lnQD0)/Δt

(QD indicates ground diameter, QD0 indicates the ground diameter on day 0, and Δt indicates salt stress days)

#### 4.3.2. The Relative Water Content (RWC)

Following the method outlined in another study [102], fresh leaves were weighed as W1 and soaked in water for 24 h to obtain expanded weight (W2). The samples were then oven-dried at 105 °C for 20 min, followed by 80 °C until a constant weight (W3) was achieved. Relative water content (RWC) was calculated using the formula

(1) RWC=(W1−W3)/(W2−W3)×100%

#### 4.3.3. Malondialdehyde (MDA)

Utilizing a method from another study [26], the MDA content in the leaves was determined by reacting with thiobarbituric acid, and absorbance changes at 450 nm, 532 nm, and 600 nm were measured using a UV–vis spectrophotometer (Beijing Purkinje General Instrument Co., Ltd., Beijing, China). The results were expressed in µmol/g.

#### 4.3.4. Osmotic Adjustment Substances

The proline (Pro) content in leaf tissue was determined using Suolaibao assay kits and the microplate method. The SS content in the leaves was determined following the method described in another study [103]. The change in absorbance was recorded at 450 nm using a spectrophotometer, and the results were expressed in mg/g. The SP content was measured according to the Coomassie Brilliant Blue G-250 method of coloration, using the method described in another study [102]. The change in absorbance at 595 nm was recorded using a UV–vis spectrophotometer (Beijing Purkinje General Instrument Co., Ltd., Beijing, China) and expressed in mg/g.

#### 4.3.5. Antioxidant Substances

SOD activity was determined following the method described in another study [23]. The absorbance change at 560 nm was measured on a UV–vis spectrophotometer (Beijing Purkinje General Instrument Co., Ltd., Beijing, China) and reported in U/g. APX activity was measured following a previously described method [104], with OD_290_ kinetics analyzed via a UV–vis spectrophotometer (Beijing Purkinje General Instrument Co., Ltd., Beijing, China) using a specified kinetics program. The reaction rate was determined based on the 10 s time point. Molar absorptivity was set at 2.8 mM/cm, expressing the results in U/g. The TFC measurements were conducted following a previously described method [105]. Absorbance changes at 760 nm were noted on a UV–vis spectrophotometer (Beijing Purkinje General Instrument Co., Ltd., Beijing, China) and reported in mg/g. TPC was measured following a previously described method [106]. Absorbance changes at 510 nm were recorded on a UV–vis spectrophotometer (Beijing Purkinje General Instrument Co., Ltd., Beijing, China) and reported in mg/g.

#### 4.3.6. Micromorpho-Anatomical Characteristics

Leaf anatomy was examined as per another study [107]. The middle sections of mature functional leaves were sampled, fixed in FAA (70% ethanol 90 mL + formaldehyde 5 mL + acetic acid 5 mL), dehydrated using ethanol and xylene, embedded in paraffin, and sliced into 8 µm thick sections using a LEICA RM 2245 (Leica Instruments Co., Ltd., Wholesaler, UK). The sections were stained with TBO and photographed under a microscope (Beijing Deep Field Technology Co., Ltd., Beijing, China), with 15 fields of view examined for each sample. Image processing was performed using Image J 1.53t (Wayne Rasband and National Institutes of Health, USA) software.

Root anatomy was examined following the method outlined in another study [108]. The samples were taken from the first-order lateral root, 3 cm from the main root. These samples were fixed with FAA, dehydrated using ethanol and xylene, embedded in paraffin, and sliced into 10 µm thick sections using a LEICA RM 2245 (Leica Instruments Co., Ltd., Wholesaler, UK). Thin sections were photographed under an optical microscope (Beijing Deep Field Technology Co., Ltd., Beijing, China), with 15 fields of view examined per sample. Image processing was conducted using ImageJ 1.53t software (Wayne Rasband and National Institutes of Health, USA).

### 4.4. Statistical Analysis

Microsoft Excel 2021, version 2309 (Microsoft Corp., Washington, DC, USA), was employed for preliminary data exploration. IBM SPSS Statistics 26.0, version 26 (International Business Machines Corp., Armonk, NY, USA), was utilized for one-way ANOVA to identify significant differences among parameters. The Duncan test, with a significance level of *p* < 0.05, facilitated mean separation. Two-way ANOVA of variance examined the effects of varying stress durations, NaCl concentrations, and their interactions. The results were reported as mean ± SD. A correlation study investigating growth, physiology, and anatomy under salt stress was conducted using the Pearson correlation at a probability level of *p* ≤ 0.05, and the correlation coefficient graph was generated using Origin 2022. Ward’s method as the amalgamation rule and the squared Euclidean distance as metric were used to establish clusters. Principal component analysis (PCA) was executed using SPSS to uncover optimal relationships among salt stress and experimentally measured characteristics, and the principal component change graph was constructed using Origin 2022.

## 5. Conclusions and Perspectives

**Figure 12:**
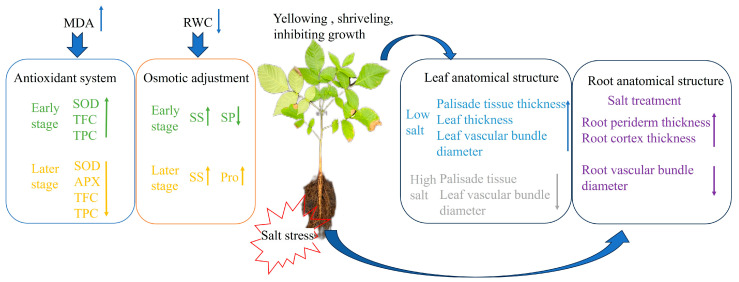
Salt tolerance model of hybrid walnut.

Based on the results of the analysis of seedling growth, physiological and biochemical characteristics, and anatomical structure to salt stress, it was found that the effect on seedlings was minimal under a low salt concentration (50 mM), while it was strongly inhibited under a high salt concentration (100, 200 mM). The hybrid walnut demonstrated a mitigating effect on the damage caused by saline stress by increasing the concentration of osmoregulatory substances and the activity of antioxidant enzymes. Seedling roots adapted to salt stress by increasing the thickness of the cortex and reducing the diameter of the vascular bundle. Leaf anatomy was promoted under low salt concentrations, and severe damage was caused by long-term high salt concentrations. Based on comprehensive analysis, SOD, TPC, Rd, SS, spongy tissue thickness, SR, and root diameter were chosen as indicators for assessing salt tolerance in hybrid walnut (*Juglans major* × *Juglans regia*) seedlings. This study provides a theoretical foundation for hybrid walnut salt tolerance and technical backing for expanding walnut cultivation indices for evaluating salt tolerance in the seedlings of hybrid walnut (*Juglans major* × *Juglans regia)* (Figure 12).

However, it should be noted that this study only considered the influence of NaCl, and the compound saline–alkali stress is more in line with the actual situation. The greenhouse experiment aims to simulate the field saline soil scenario with peat as the substrate, but it is necessary to conduct large-scale field experiments, which are essential for further expanding the walnut planting area. In addition, our next research focuses on discovering Na^+^ receptors and other components involved in the regulation of ion transporters in walnuts. At the same time, using genomics, transcriptomics, proteomics, metabolomics, and epigenomics, we further investigated the key genes in plant response to salt stress and analyzed the signaling mechanism in response to salt stress to provide a theoretical basis and key targets for the cultivation of salt-tolerant walnut rootstocks.

## Data Availability

Data are contained within the article.
